# Early screening model for mild cognitive impairment based on resting-state functional connectivity: a functional near-infrared spectroscopy study

**DOI:** 10.1117/1.NPh.9.4.045010

**Published:** 2022-12-06

**Authors:** Shen Zhang, Ting Zhu, Yizhu Tian, Wenyu Jiang, Deyu Li, Daifa Wang

**Affiliations:** a Beihang University, School of Biological Science and Medical Engineering, Beijing Advanced Innovation Centre for Biomedical Engineering, Key Laboratory for Biomechanics and Mechanobiology of Ministry of Education, Beijing, China; b Guangxi Jiangbin Hospital, Department of Neurological Rehabilitation, Nanning, China; c Beihang University, State Key Laboratory of Software Development Environment, Beijing, China; d Beihang University, State Key Laboratory of Virtual Reality Technology and System, Beijing, China

**Keywords:** functional near-infrared spectroscopy, amnesic mild cognitive impairment, resting state, functional connectivity

## Abstract

**Significance:**

As an early stage of Alzheimer’s disease (AD), the diagnosis of amnestic mild cognitive impairment (aMCI) has important clinical value for timely intervention of AD. Functional near-infrared spectroscopy (fNIRS)-based resting-state brain connectivity analysis, which could provide an economic and quick screening strategy for aMCI, remains to be extensively investigated.

**Aim:**

This study aimed to verify the feasibility of fNIRS-based resting-state brain connectivity for evaluating brain function in patients with aMCI, and to determine an early screening model for auxiliary diagnosis.

**Approach:**

The resting-state fNIRS was utilized for exploring the changes in functional connectivity of 64 patients with aMCI. The region of interest (ROI)-based and channel-based connections with significant inter-group differences have been extracted through the two-sample t-tests and the receiver operating characteristic (ROC). These connections with specificity and sensitivity were then taken as features for classification.

**Results:**

Compared with healthy controls, connections of the MCI group were significantly reduced between the bilateral prefrontal, parietal, occipital, and right temporal lobes. Specifically, the long-range connections from prefrontal to occipital lobe, and from prefrontal to parietal lobe, exhibited stronger identifiability (area under the ROC curve >0.65, **p<0.01). Subsequently, the optimal classification accuracy of ROI-based connections was 71.59%. Furthermore, the most responsive connections were located between the right dorsolateral prefrontal lobe and the left occipital lobe, concomitant with the highest classification accuracy of 73.86%.

**Conclusion:**

Our findings indicate that fNIRS-based resting-state functional connectivity analysis could support MCI diagnosis. Notably, long-range connections involving the prefrontal and occipital lobes have the potential to be efficient biomarkers.

## 1 Introduction

Alzheimer’s disease (AD) is the most common neurodegenerative disorder among the elderly. It damages brain cells and nerves, and disturbs the storage and transmission of information in the brain. It is even potentially fatal without timely intervention. According to the statistics of the World Health Organization, more than 9.9 million new cases of dementia are diagnosed worldwide every year, of which about 60% to 70% are classified as AD.^1^ Although scientists agree that the disease is associated with abnormalities of certain specific proteins (beta-amyloid and tau) in the brain, unfortunately the cause of AD is still unclear, and about 70% of the risks are considered hereditary. Mild cognitive impairment (MCI) is usually recognized as the prodromal stage of AD. Annually, MCI patients tend to convert to AD with a clinical conversion rate higher than 10%, while it is only 1% to 2% in healthy controls.^2^^,^^3^ In amnestic MCI (aMCI), the conversion rate is even higher. Therefore, early identification of MCI is crucial for timely intervention of potential AD patients. More importantly, with the acceleration of aging, professional doctors for AD are in short supply, so fast and economical screening methods suitable for large population are increasingly needed.

Functional connectivity analysis has been widely used in the evaluation of brain function.^4^^,^^5^ The human brain is structurally a complex network composed of a large number of neurons and nerve fibers, and various regions of the brain are interrelated..^6^^,^^7^ Some synchronous low-frequency fluctuations are associated with neural activity between some brain regions at even resting state, which indicates that organized activities between different brain regions contribute to maintaining the mechanism of brain activity.^8^ AD is increasingly viewed as a disease with multiple dysfunctional large-scale neuronal networks rather than a localized abnormality, which makes it feasible to detect the brain anomalies from the perspective of resting-state functional connectivity (rs-FC). The rs-FC analysis has received increasing attention in the subtle network abnormalities of AD or MCI. Sheline et al.^9^ and Oh et al.^10^ applied functional magnetic resonance imaging (fMRI)-based rs-FC to reveal that, the connectivity of the default mode network in cognitively normal elderly is significantly reduced when beta-amyloid increases. Drzezga et al.^11^ reported that the whole-brain connectivity was positively correlated with metabolism and negatively correlated with amyloid burden. Similarly, Wang et al.^12^ and Liu et al.^13^ adopted fMRI-based rs-FC to determine the abnormal functional connectivity in patients with MCI. Reduced connection has been detected in the regions of left dorsolateral superior frontal gyrus, the right orbital frontal gyrus and left inferior temporal gyrus.

Functional near-infrared spectroscopy (fNIRS) is a new non-invasive brain imaging technology with unique advantages in clinical application, such as high portability and low running cost, relative robust-ness against motion and electrical artifacts. Compared with some existing classical brain imaging technologies like fMRI, EEG, or PET, fNIRS is more friendly to the subjects and more suitable for monitoring under clinical conditions, which has aroused the interest of many brain function researchers. Niu et al.^14^ and Li et al. have manifested that the brain FC and related graph metrics have high stability in resting state. Tan et al.^15^ applied fNIRS-based rs-FC to explore synchronization of blood oxygen signals between bilateral prefrontal cortex, they found the brain FC of the elderly are weaker than the young in certain frequency bands.

fNIRS has also been used to explore the blood oxygen level dependence of patients with MCI or AD. Viola et al.^16^ reported that tissue oxygenation of prefrontal lobe and temporal parietal cortex in aMCI patients was lower than that in healthy control group. In 2014, Liu et al.^17^ found that the cerebral blood flow and cerebral blood oxygen metabolism of aMCI patients were lower than those of healthy people. Moreover, some task-related experiments were also used to determine changes in brain function, of which word retrieval is the most commonly used one. Yeung and Chan^18^ found that the activation of frontal temporal lobe or parietal cortex in aMCI patients was less, and the lateral response of prefrontal lobe also changed. Zeller et al.^19^ analyzed spontaneous low frequency oscillations (LFO) with fNIRS and observed a decreased LFO of the parietal cortex for aMCI group. As far as we know, only three fNIRS studies involve changes in functional connectivity in MCI patients. Niu et al.^14^ detected the brains of MCI and AD in the resting state. Although abnormal dynamic FCs were obtained in 23 patients, no differences between groups were found in the resting state FC. Nguyen et al.^20^ used four monitoring channels to record FC of aMCI patients and reported changes in internal and internal connections in the cerebral hemisphere of aMCI patients. Bu et al.^21^ investigated the resting-state effective connectivity (EC) in 26 aMCI patients and claimed a significantly decreased EC among prefrontal, motor, and occipital cortex.

To sum up, fNIRS-based rs-FC analysis has the potential to provide economic and fast screening strategies for MCI. It not only requires less preparation and monitoring period, but also is suitable for cognitive impairment patients who cannot complete complex tasks well. Thus, clinicians could record functional data at various stages of MCI, which is crucial for early diagnosis of the disease and evaluation of drugs and therapeutic effects. However, at present, effective biological markers based on fNIRS rs-FC need further exploration. In view of this, we aimed to explore an early screening model for MCI. Specifically, the resting-state fNIRS was utilized for determining the changes of functional connectivity of 64 patients with MCI. According to the significant differences of functional connectivity between aMCI and HC groups, the cortical region of lesions induced by aMCI has been extracted as a critical area for diagnosis. On this basis, we have highlighted the most sensitive cortical position and suggested a simpler screening strategy.

## 2 Materials and Method

### 2.1 Participants

With the help of the professional neurologists and the community hospital, 150 participants were recruited in this study. After excluding the participants who did not complete the experiment or whose data quality did not meet the requirements [channel signals with signal-to-noise ratio (SNR) <10  dB], a total of 128 samples were used for further analysis, including 64 patients with aMCI and 64 healthy controls. All the participants were assessed by the mini-mental state examination (MMSE) and the Montreal cognitive assessment (MoCA), which are brief cognitive screening tools that have been developed for the screening of patients with MCI. Independent sample t-tests were run to examine group differences in age and neuropsychological scores, and the gender proportions were analyzed using the $\chi^{2}$ test. The specific scores and demographic information of the subjects were given in Table 1. There was no significant difference in age and gender between HC and aMCI groups, but the scale scores were on the contrary. This study was conducted according to the declaration of Helsinki and approved by the local Ethics Committee of Beihang University.

**Table 1:** Socio-demographic information for the present study.

	HC	aMCI	p
Age	66.98±5.23	67.45±4.91	0.60
Gender	37M/27F	29M/35F	0.16
MMSE	27.72±1.65	25.31±2.43	<0.01
MoCA	25.23±2.1	19.72±1.84	<0.01

### 2.2 Data Acquisition and Preprocessing

The fNIRS signals were acquired using a multichannel fNIRS system (NirScan-8000A, HuiChuang, China) with two wavelengths (730 nm, 850 nm) at a sampling rate of 19 Hz. It supports the automatic adjustment of the source power and detector gain to optimize signal quality.

**Fig. 1:**
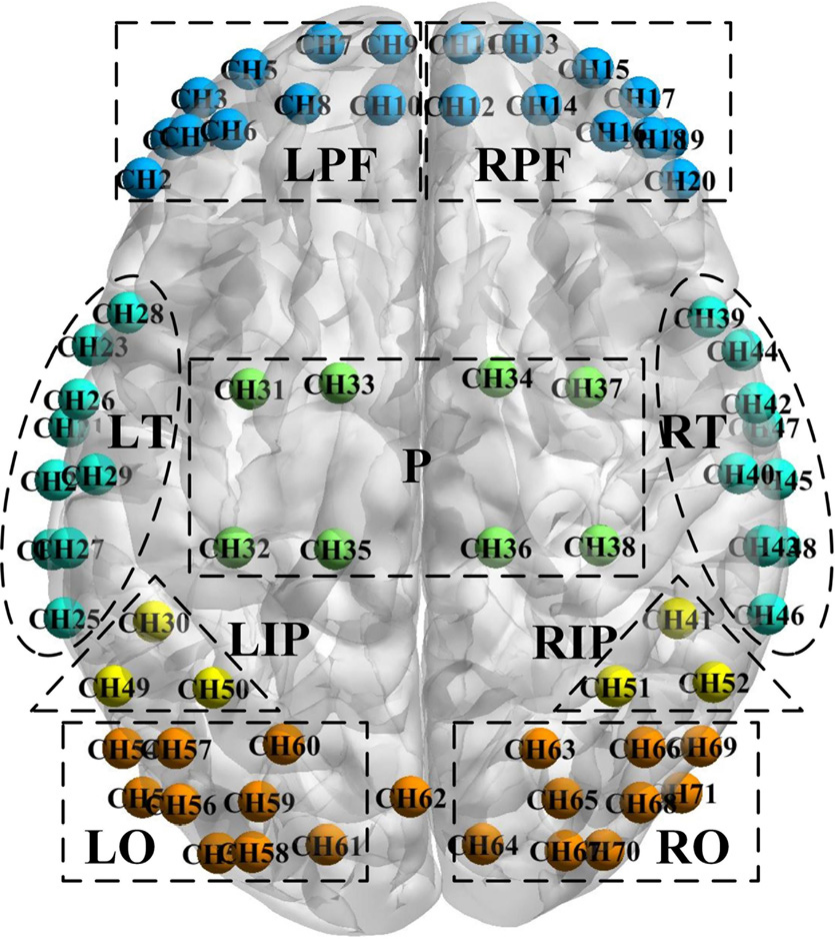
The arrangement of measuring channels. Each ball represented a channel (CH), and the color indicated which region the channel belongs to. The nine ROIs were as follow: LPF = left prefrontal lobe; RPF = right prefrontal lobe; LT = left temporal lobe; P = superior parietal lobe; RT = right temporal lobe; LIP = left inferior parietal lobe; RIP = right inferior parietal lobe; LO = left occipital lobe; and RO = right occipital lobe.

The SNR was evaluated from the time series d (raw light intensity, d>0) of each measurement channel, where the mean of d was considered as “signal,” and the standard deviation of d was considered as “noise.” Then SNR was calculated as follows:^22^ SNR = mean (d)/std (d). Subsequently, the SNR values were converted to dB by a logarithmic operation. The average SNR of channels used was 39.2±14.5  dB. The arrangement of measuring channels covered the prefrontal lobe, temporal lobe, parietal lobe, and occipital lobe of the brain, consisted of 22 sources and 31 detectors. The 71 measurement channels were grouped into nine anatomic macro-areas for further analysis (see Fig. 1). A three-dimensional digitizer was utilized to measure the spatial location of each optode for all participants. Then the NIRS-SPM software was used to access each channel’s mean Montreal Neurological Institute (MNI) standard coordinates,^23^ which were given in Table 2.

**Table 2:** The mean MNI coordinates and anatomical labels corresponding to the measurement channels.

No.	MNI coordinates			Region of interest	AAL	No.	MNI coordinates			Region of interest	Anatomic label (AAL)
	x	y	z				x	y	z		
1	−46.4	47.9	−4.7	LPF	Inferior frontal gyrus	37	16.0	2.4	73.1	P	Middle frontal gyrus right
2	−51.7	40.4	10.6	LPF	Superior frontal gyrus (dorsal) left	38	−15.0	−30.6	77.3	P	Inferior parietal right
3	−40.9	55.7	3.2	LPF	Inferior frontal gyrus	39	15.8	−30.3	78.0	RT	Superior temporal gyrus right
4	−43.2	48.6	18.7	LPF	Superior frontal gyrus (dorsal) left	40	53.1	0.3	50.2	RT	Inferior frontal gyrus (opercular)
5	−31.4	61.2	4.9	LPF	Middle frontal gyrus left	41	55.7	−29.9	54.1	RIP	Angular gyrus right
6	−35.4	49.7	27.7	LPF	Middle frontal gyrus left	42	56.5	13.8	33.8	RT	Temporal pole (superior) right
7	−17.1	66.1	6.9	LPF	Middle frontal gyrus left	43	62.3	−16.1	40.1	RT	Heschl gyrus right
8	−21.2	54.6	29.8	LPF	Middle frontal gyrus left	44	60.6	−43.8	41.7	RT	Temporal pole (superior) right
9	−4.6	66.1	8.7	LPF	Superior frontal gyrus (medial)	45	65.6	−2.6	23.6	RT	Middle temporal gyrus right
10	−5.4	54.4	34.9	LPF	Superior frontal gyrus (medial)	46	67.1	−30.0	27.7	RT	Middle temporal gyrus right
11	9.6	66.1	8.7	RPF	Superior frontal gyrus (medial)	47	63.0	7.5	3.4	RT	Middle temporal gyrus right
12	8.8	54.3	35.0	RPF	Superior frontal gyrus (medial)	48	69.6	−17.2	7.7	RT	Middle temporal gyrus right
13	21.0	66.3	6.7	RPF	Middle frontal gyrus right	49	67.9	−43.0	11.4	LIP	Angular gyrus left
14	24.5	54.4	30.3	RPF	Middle frontal gyrus right	50	67.0	−7.1	−12.5	LIP	Angular gyrus left
15	34.4	61.4	4.5	RPF	Middle frontal gyrus right	51	70.3	−30.2	−8.6	RIP	Angular gyrus right
16	37.8	49.4	28.1	RPF	Middle frontal gyrus right	52	−57.3	−56.8	42.4	RIP	Angular gyrus right
17	43.3	55.8	3.3	RPF	Superior frontal gyrus (dorsal) right	53	−38.3	−57.1	63.1	LO	Cerebellum_Crus1 left
18	45.5	48.1	19.3	RPF	Inferior frontal gyrus	54	38.7	−56.8	63.2	LO	Cerebellum_Crus1 left
19	48.8	47.9	−4.3	RPF	Superior frontal gyrus (dorsal) right	55	57.5	−56.0	43.3	LO	Inferior occipital left
20	51.0	40.1	11.7	RPF	Inferior frontal gyrus	56	−51.8	−78.1	0.5	LO	Middle occipital left
21	−65.9	−7.1	−13.7	LT	Middle temporal gyrus left	57	−55.6	−68.7	27.2	LO	Inferior occipital left
22	−69.2	−30.7	−9.4	LT	Middle temporal gyrus left	58	−37.4	−88.7	18.8	LO	Middle occipital left
23	−61.4	8.4	2.4	LT	Temporal pole (superior) left	59	−44.6	−79.4	34.7	LO	Middle occipital left
24	−68.5	−17.3	7.5	LT	Middle temporal gyrus left	60	−45.5	−68.7	48.8	LO	Superior occipital left
25	−66.8	−43.7	11.2	LT	Middle temporal gyrus left	61	−30.8	−88.5	34.9	LO	Middle occipital left
26	−64.0	−1.9	23.7	LT	Temporal pole (superior) left	62	−29.6	−78.9	45.6	MO	Superior occipital (medial)
27	−66.1	−30.4	28.1	LT	Heschl gyrus left	63	−24.8	−67.9	63.4	RO	Superior occipital right
28	−54.7	14.6	33.0	LT	Superior temporal gyrus left	64	−16.6	−87.1	37.4	RO	Middle occipital right
29	−60.7	−16.3	40.5	LT	Inferior frontal gyrus (opercular)	65	−0.5	−78.0	56.8	RO	Middle occipital right
30	−59.7	−44.4	42.1	LIP	Angular gyrus left	66	24.2	−68.7	64.0	RO	Inferior occipital right
31	−51.4	0.1	49.9	P	Middle frontal gyrus left	67	13.3	−87.0	36.9	RO	Middle occipital right
32	−54.3	−30.3	54.5	P	Inferior parietal left	68	28.5	−78.3	45.8	RO	Middle occipital right
33	−46.4	47.9	−4.7	P	Middle frontal gyrus left	69	44.3	−68.5	48.9	RO	Cerebellum_Crus1 right
34	−51.7	40.4	10.6	P	Middle frontal gyrus right	70	29.1	−88.2	34.0	RO	Inferior occipital right
35	−40.9	55.7	3.2	P	Superior parietal left	71	43.5	−79.1	34.6	RO	Cerebellum_Crus1 right
36	−14.2	1.2	72.9	P	Superior parietal right						

Note: MO = middle occipital lobe.

The experiment was performed in a confined and moderately dim room to reduce any disturbance to the participants from the environment. During the experiment, subjects were seated in a comfortable chair and were required to stay relaxed without any extra movement. Subsequently, 10 min of resting-state data were collected for each subject.

The NirSpark (HuiChuang, China) was applied to preprocess obtained signals. Motion artifacts affect functional connectivity analysis deeply and has attracted much attention.^24^^–^^26^ The moving standard deviation and spline interpolation methods^24^ (SDThresh = 20, AMPThresh = 3, tMotion = 0.5s, tMask = 1s and *p* = 0.99) were applied to amend motion artifacts caused by the relative sliding of the scalp and probes. Subsequently, 0.01 to 0.1 Hz bandpass filtering was performed to remove systematic physiological noises such as pulse and respiration.^27^ Then the modified Beer–Lambert law was used to transform light intensity into the relative change of hemoglobin concentration. The differential path-length factor was set to 6.0.^28^^,^^29^ In 2007, Hoshi^30^ investigated the relationship between NIRS signals and cortical blood flow (CBF) through a developed perfused rat brain mode. He confirmed that oxygenated hemoglobin (HbO) is more consistent with CBF variations than deoxygenated hemoglobin (HbR) and is a more sensitive indicator in NIRS measurements. Similarly, HbO was reported to have a relatively higher SNR than HbR in subsequent studies.^31^^,^^32^ Thus, we have presented the following result through the time series of HbO signals.

### 2.3 Brain Functional Connectivity

The Pearson’s correlation coefficient has been calculated to determine the functional connection between each pair of measurement channels. Thus, a 71×71 correlation matrix would be generated for each participant. Subsequently, Fisher’s r-to-z transformation was applied to convert these correlation coefficients to z-scores for improved normality.

Changes in functional connectivity were observed from three perspectives: whole-brain averaged, region of interest (ROI)-based, and channel-based. In ROI analysis, 71 measurement channels were divided into nine brain regions based on their location (see Table 2), including the left prefrontal lobe (LPF), right prefrontal lobe (RPF), left temporal lobe (LT), superior parietal lobe (P), right temporal lobe (RT), left inferior parietal lobe (LIP), right inferior parietal lobe (RIP), left occipital lobe (LO), and right occipital lobe (RO). Then the time series of the nine ROIs’ internal channels were averaged to obtain ROI-based z-scores.

### 2.4 Statistical Analysis

The two-sample t-tests and false discovery rate (FDR) correction were employed to compare differences in functional connections between the MCI and HC groups. Seventy-one channels constituted (71×70/2)=2485 undirected connections for each subject. Taking channel-based analysis as an example, the unpaired t-tests were used to calculate group differences for these connections, resulting in a total of 2485 modified p values after FDR correction. Where p values <0.05 were considered to be significantly different (*p<0.05), while <0.01 were considered to be extremely significantly different (**p<0.01).

Furthermore, the receiver operating characteristic (ROC) curve approach was adopted to assess the sensitivity and specificity of these differential connections. The area under the ROC curve (AUC) was conducted to quantify the performances of these features in identifying MCI.

Afterwards, connections with significant differences were extracted as features input to a linear discriminant analysis (LDA) for classification. Based on the AUC values and modified p values, six ROI-based (AUC>0.65) and four channel-based (**p<0.01), individually or in combination, participated in the model training. Then 5-fold cross-validation classification accuracies were reported.^33^^,^^34^ Specifically, 62 MCI samples were randomly split into training and validation set (4:1), as well as the HC group. To reduce the uncertainty of the random component and improve the stability of the classification performance, the above steps were repeated N=10 times and averaged before presentation.

## 3 Result

### 3.1 Whole-Brain Mean Functional Connectivity

**Fig. 2:**
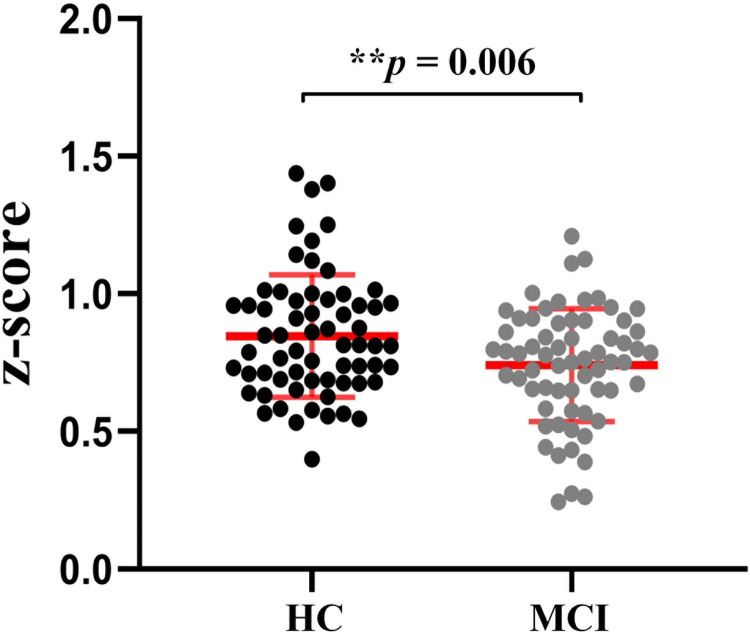
The inter-group differences in whole-brain mean functional connectivity. Black and grey points represent the HC and MCI groups, respectively. Asterisk indicates an extremely significant difference (**p<0.01).

To verify the effect of MCI on functional connectivity of the whole brain, all the 2485 connections (z-scores) were averaged for each participant. Then two-sample t-tests were applied to calculate the inter-group differences, as shown in Fig. 2. Compared with the HC group, the MCI group has significantly weakened [t(126)=2.79, **p<0.01] functional connections (mean±SD: 0.85±0.22 and 0.74±0.21, respectively).

### 3.2 ROI-Based Functional Connectivity

**Fig. 3:**
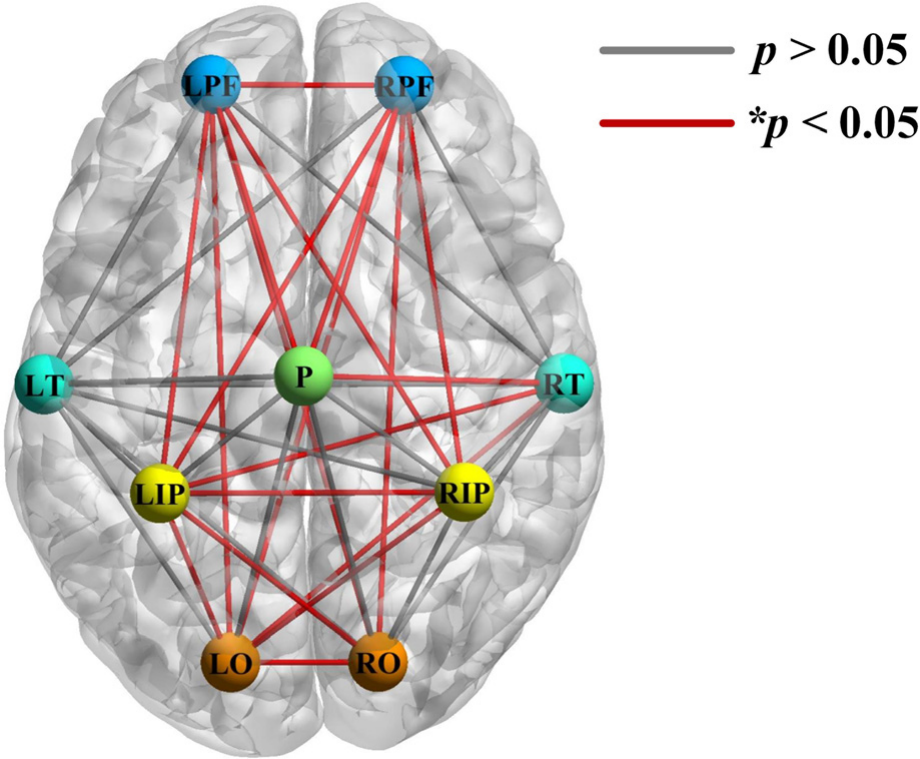
Regional differences in functional connectivity. Red lines indicate significant inter-group differences in the regional connection.

All 71 channels were divided into nine ROIs to further explore the between-ROI connectivity characteristics. Two-sample t-tests and FDR correction were applied to extract critical areas lesions induced by MCI. There were 19 connections with significant inter-group differences (*p<0.05), most of which involved the bilateral prefrontal, occipital, and inferior parietal lobes, as shown in Fig. 3.

**Fig. 4:**
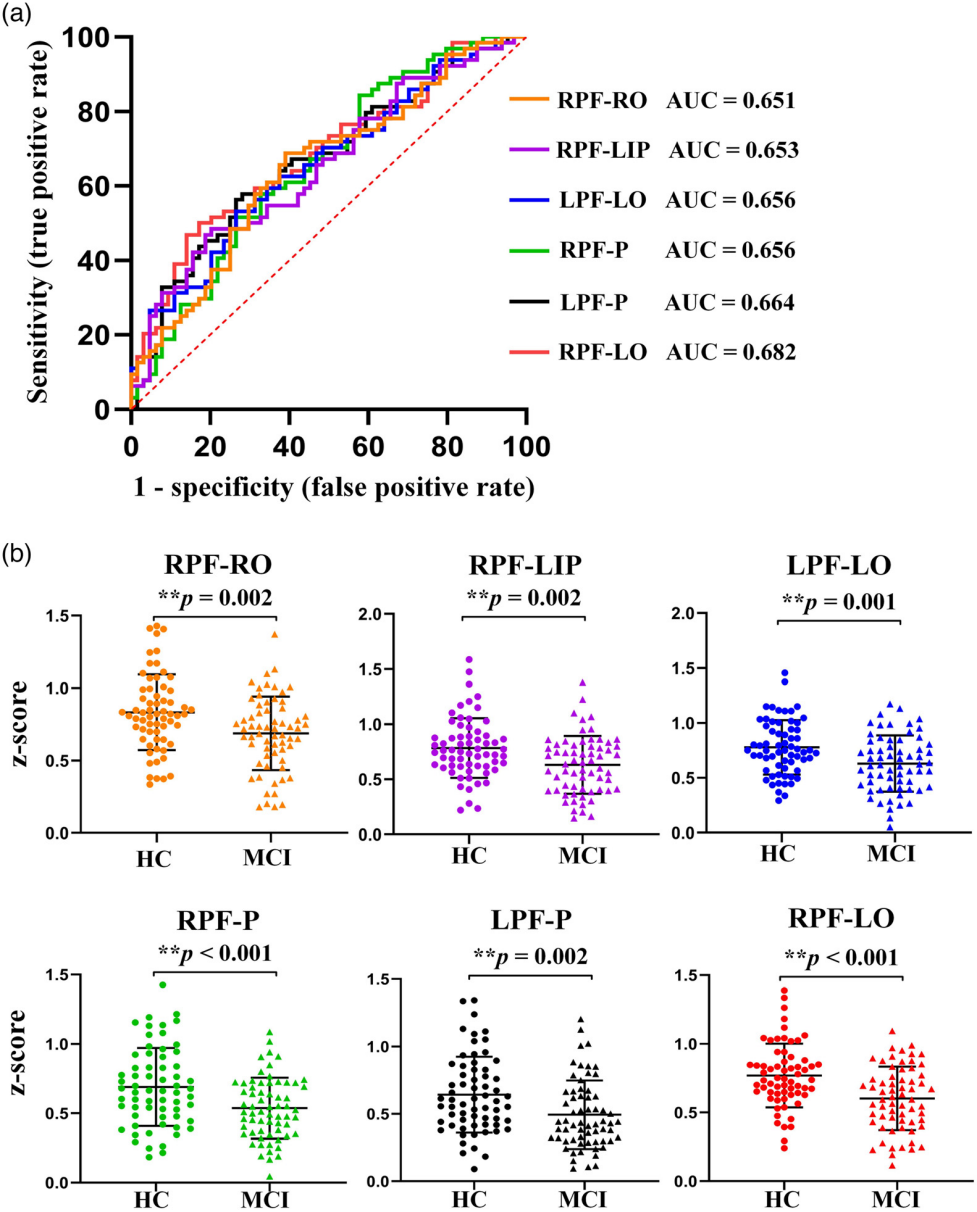
(a) The ROC curves of the top six long-range connections in ascending order: RPF-RO (orange), RPF-LIP (purple), LPF-LO (blue), RPF-P (green), LPF-P (black), and RPF-LO (red). (b) The inter-group differences in the top six ROI-based functional connections. The color settings of the connections are consistent with (a). Circles and triangles represent the HC and MCI groups, respectively. Asterisk indicates an extremely significant difference (**p<0.01).

To further identify the variation of long-distance connections in patients with MCI, the ROC analysis was carried out to determine the sensitivity and specificity of 19 connections. Specifically, the higher AUC value represents a better balance between specificity and sensitivity, which means that it may have better recognition performance for MCI. Figure 4(a) shows the results of the top six long-distance connections in ascending order: RPF-RO, RPF-LIP, LPF-LO, RPF-P, LPF-P, and RPF-LO (the AUC values are 0.651, 0.653, 0.656, 0.656, 0.664, and 0.682, respectively).

The group differences in the top six ROI-based functional connections are shown in Fig. 4(b). Compared with the HC group, the MCI group exhibited significantly reduced cross-interval brain functional connectivity in RPF-RO [MCI: 0.69±0.25; HC: 0.83±0.26; t(126)=3.19, **p=0.002], RPF-LIP [MCI: 0.63±0.26; HC: 0.78±0.27; t(126)=3.22, **p=0.002], LPF-LO [MCI: 0.63±0.26; HC: 0.78±0.25; t(126)=3.31, **p=0.001], RPF-P [MCI: 0.54±0.22; HC: 0.69±0.28; t(126)=3.42, **p<0.001], LPF-P [MCI: 0.49±0.25; HC: 0.64±0.28; t(126)=3.14, **p=0.002], and RPF-LO [MCI: 0.60±0.23; HC: 0.77±0.23; t(126)=4.08, **p<0.001].

### 3.3 Channel-Based Functional Connectivity

**Fig. 5:**
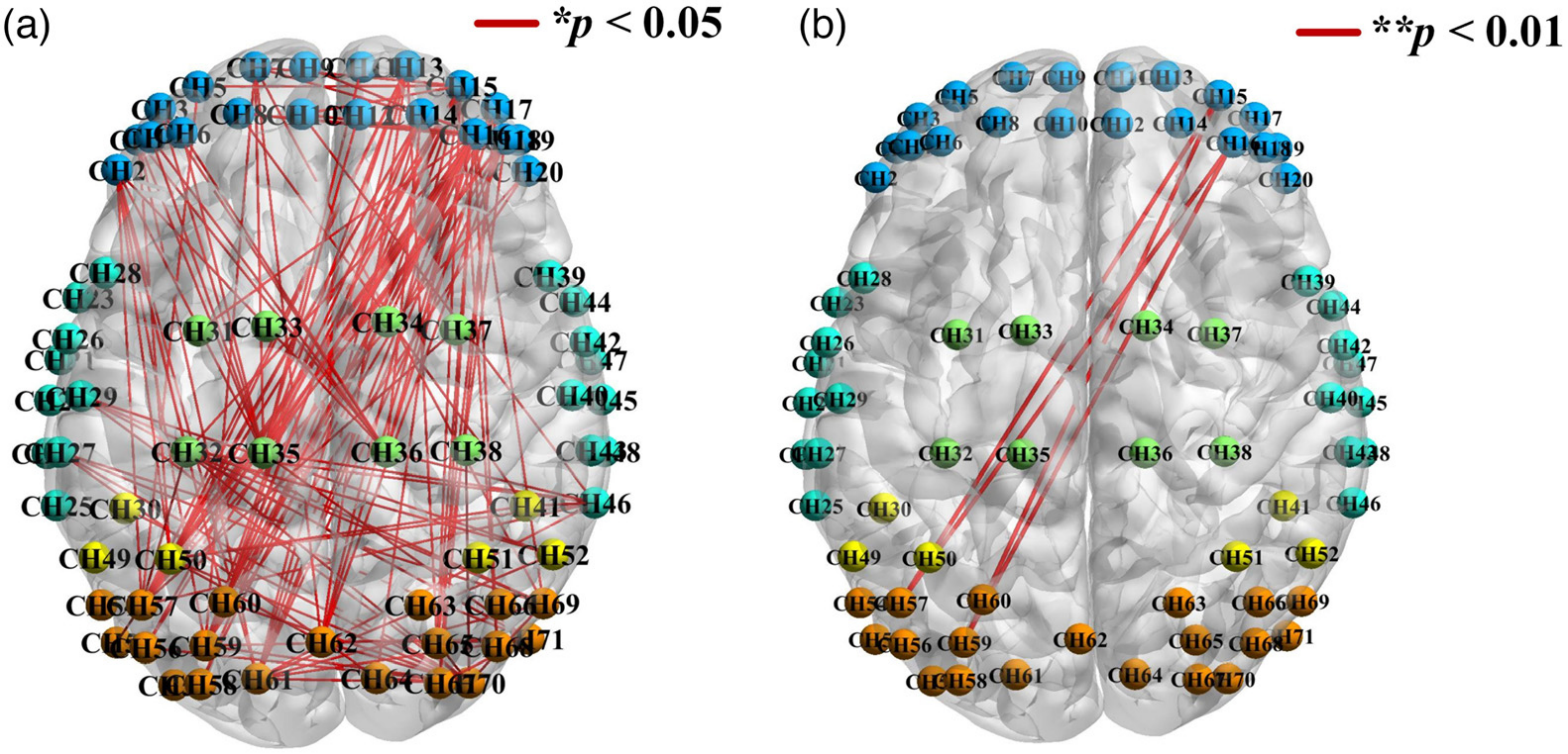
(a) Channel-based connections with inter-group differences (*p<0.05). (b) Connections with highly significant difference (****p<0.01).

Similarly, the inter-group differences in channel-based FCs between groups were also calculated by t-tests and FDR correction. As shown in Fig. 5(a), a total of 138 connections had significant inter-group differences. Moreover, in order to confirm the most sensitive cortical location and reduce the feature dimension for classification, four connections with p values <0.01 were screened out Fig. 5(b)]. Specifically, they were CH15-CH57 (sensitivity = 63.6%, specificity = 67.1%), CH16-CH59 (sensitivity = 59.1%, specificity = 70.5%), CH16-CH57 (sensitivity = 61.4%, specificity = 68.2%), CH15-CH59 (sensitivity = 67.2%, specificity = 70.3%), respectively, and all belonged to the connection between the right dorsolateral prefrontal lobe and the left occipital lobe.

### 3.4 Classification

For the need of clinical diagnosis, the effective prediction of MCI through a small number of monitoring channels will help to improve the screening efficiency. Based on the AUC values and modified p values, six ROI-based (AUC>0.65) and four channel-based (**p<0.01) participated in the model training.

The classification accuracies are given in Table 3. RPF-LO performed best for the ROI-based classification, and was consistent with AUC values. The optimal classification accuracy was 71.59%, and the mean classification accuracy was 66.48±2.79%. For the channel-based classification, CH15-CH59 contributed comparatively desirable performance with an optimal classification accuracy of 73.86% and an average classification accuracy of 68.41±2.82%.

**Table 3:** The classification accuracies of ROI-based and channel-based connections.

N	1	2	3	4	5	6	7	8	9	10	Mean value
ROI-based											
RPF-RO	67.05%	64.77%	61.36%	65.91%	62.50%	67.05%	62.50%	67.05%	65.91%	62.50%	64.66±2.13%
RPF-LIP	60.23%	62.50%	61.36%	63.64%	62.50%	67.05%	62.50%	62.50%	63.64%	69.32%	63.52±2.56%
LPF-LO	60.23%	65.91%	62.50%	64.77%	65.91%	64.77%	60.23%	68.18%	70.45%	59.09%	64.20±3.49%
RPF-P	65.91%	61.36%	60.23%	63.64%	65.91%	67.05%	61.36%	63.64%	61.36%	67.05%	63.75±2.46%
LPF-P	60.23%	67.05%	64.77%	60.23%	62.50%	63.64%	63.64%	64.77%	68.18%	65.91%	64.09±2.50%
RPF-LO	63.64%	62.50%	63.64%	67.05%	64.77%	65.91%	69.32%	71.59%	69.32%	67.05%	66.48±2.79%
All above	65.91%	65.91%	69.32%	67.05%	62.50%	67.05%	62.50%	65.91%	64.77%	59.09%	65.00±2.78%
Channel-based											
CH15-CH57	63.64%	71.59%	62.50%	60.23%	69.32%	64.77%	60.23%	64.77%	63.64%	60.23%	64.09±3.64%
CH16-CH59	62.50%	69.32%	67.05%	62.50%	64.77%	63.64%	68.18%	60.23%	63.64%	61.36%	64.32±2.84%
CH16-CH57	62.50%	71.59%	63.64%	61.36%	68.18%	61.36%	62.50%	68.18%	69.32%	63.64%	65.23±3.53%
CH15-CH59	69.32%	65.91%	65.91%	67.05%	71.59%	64.77%	65.91%	73.86%	69.32%	70.45%	68.41±2.82%
All above	67.05%	69.32%	63.64%	68.18%	65.91%	63.64%	64.77%	62.50%	68.18%	64.77%	65.80±2.18%

## 4 Discussion

In the present study, we explored the changes of functional connectivity of 64 patients with MCI. Ulteriorly, relatively sensitive connections were extracted to identify MCI at both channel- and ROI- levels.

For the whole-brain perspective, the MCI group had significantly reduced functional connectivity (**p<0.01), indicating that cognitive decline is widespread across the brain. It may be related to hypoperfusion and hypometabolic patterns in MCI patients. In 2015, Li et al.^35^ reported significant hypoperfusion in the prefrontal cortex of MCI patients using a meta-analysis of 39 fMRI-based studies. Similarly, Coutinho et al.^36^ found hypoperfusion in the prefrontal and temporal lobes of MCI patients, which was more pronounced in AD patients. This cerebrovascular dysfunction is likely to result in weaker low-frequency blood flow turbulence, reduce tissue oxygenation levels in the cerebral cortex, and trigger a decline in global cerebral synergistic capacity. Based on these phenomena, researchers^16^^,^^37^ have come to believe that the abnormalities in functional connectivity may potentially aid in identifying MCI.

With respect to the ROI-based results, there were severe losses of long-range connections between the brain regions of patients with MCI. Specifically, connections were significantly reduced between the bilateral prefrontal, parietal, occipital, and right temporal lobes. It implies that MCI induces a decrease in the efficiency of information exchange between various parts of cortical areas, with clinical manifestations of diminished brain coordination. The prefrontal cortex is mainly involved in some advanced cognitive processing, while the parietal, temporal, and occipital lobes are responsible primarily for the processing of sensory, auditory, and visual information. The diminished communication between these brain regions is consistent with clinical symptoms of MCI patients, including dysfunction of executive function, attention, visuospatial functions, and memory. In 2012, Brier et al.^38^ have reported the loss of functional connectivity in large-scale networks of MCI patients using rs-fMRI, including default networks and sensory motor network, etc. Bu et al.^21^ investigated functional connectivity in aMCI and found that changes were principally between the bilateral prefrontal and occipital lobes. This could be attributed to anatomical changes such as loss of white matter. For example, some previous studies^39^ have revealed that callosal degeneration in MCI patients leads to abnormal information exchange between the bilateral brains. Correspondingly, the ROI-based results mentioned above also showed a significant decrease in connectivity at corresponding locations in both cortical layers.

Notably, from the ROI-based AUC values, connections between brain regions at greater distances were more impaired (from prefrontal to occipital lobe, or from prefrontal to parietal lobe). Liang et al.^40^ evaluated the causal connections of cortical networks in aMCI patients and also found missing connections between prefrontal and inferior parietal lobules in the frontoparietal control network. Agosta et al.^41^ reported that early AD patients are characterized by loss of functional connectivity in the posterior part of the brain, particularly in the parietal lobe. Consequently, long-range connections based on rs-fNIRS hold promise as sensitive biomarkers for MCI.

For the channel-based perspective, consistent with ROI-based results, defective connections were distributed predominantly between frontal, parietal and occipital lobes. Weakness in wide-ranging connectivity again indicates degenerative changes in multiple brain regions in MCI patients; nevertheless, the extent of damage within the same region may not be consistent. As seen from the distribution of connections with deeply significant differences (**p<0.01), the most responsive connections were located between the right dorsolateral prefrontal lobe and the left occipital lobe. The right dorsolateral prefrontal cortex is associated with episodic memory, and its impairment may result in severe deterioration of self-memory, while tentorial memory loss happens to be the early symptoms in AD patients. It implies that the right dorsolateral prefrontal cortex may be one of the critical cortical lesions in MCI. We believe this may be related to cortical hypometabolism. Drzezga et al.^11^ have shown that increased amyloid deposition in cortical hubs induces a decrease in neuronal viability and hypometabolism, and spatially overlaps with the interruption of functional connectivity. Hypometabolism also further results in vasoconstriction and white matter deletion. In particular, energy is more concentrated in long-distance transmission. When white matter is injured, in order to improve energy efficiency, more effective interaction paths are selected, resulting in a weakening of long-scale connections.

To screen out features that are both sensitive and specific, we ranked ROI-based and channel-based connections by ROC analysis. Then LDA was used to train classification models, and the results showed that RPF-LO had comparatively ideal classification performance in the ROI-based perspective and could be used as a potential neural marker. Additionally, the connection between the right dorsolateral prefrontal cortex and the left occipital lobe also exhibited slightly stronger identifiability in the channel-based perspective. It means that clinicians could attain sensitive biomarkers through a small number of probe arrangements to confirm MCI rapidly and repeatedly. It opens up possibilities of large-scale MCI screening and auxiliary diagnosis. Moreover, the current conclusion still requires more samples and research to verify the stability.

In summary, MCI reduces CBF and induces structural changes, leading to a decline in the ability to collaborate between functional areas. Functional connectivity analysis based on rs-fNIRS has the potential to be a neural marker for identifying MCI, with particular concern being long-range connectivity between the prefrontal and occipital lobes. Based on our results, there are still some limitations and suggestions. In this study, we did not subdivide the different stages of aMCI. In the future, researchers could continuously track the transition of connectivity from aMCI to AD. In addition, rs-FC based on fNIRS can not only determine cortical lesions in MCI, but also support the evaluation of the effect of targeted therapy, and provide personalized rehabilitation training programs for patients.

## 5 Conclusion

In this study, fNIRS based rs-FC analysis was employed to evaluate changes in functional connectivity in MCI patients. A wide range of diminished functional connections were observed, especially the long-range ones involving the frontal, parietal, and occipital lobes. These specific and sensitive connections were considered biomarkers in an attempt to provide some support for the identification of MCI. Furthermore, the connections between the right dorsolateral prefrontal cortex and the left occipital lobe are strongly representative, deserving more attention in studies of MCI. fNIRS-based rs-FC analysis could provide economic and fast screening strategies for MCI, and it is also expected to play a unique role in the evaluation of the effect of targeted therapy in the future.
